# Training Deep Learning Based Dynamic MR Image Reconstruction Using Synthetic Fractals

**DOI:** 10.1002/mrm.70521

**Published:** 2026-07-19

**Authors:** Anirudh Raman, Olivier Jaubert, Mark Wrobel, Tina Yao, Ruaraidh Campbell, Rebecca R Baker, Ruta Virsinskaite, Daniel Knight, Michael Quail, Jennifer A Steeden, Vivek Muthurangu

**Affiliations:** ^1^ UCL Centre for Translational Cardiovascular Imaging University College London London UK; ^2^ Department of Cardiology Royal Free London NHS Foundation Trust London UK; ^3^ Institute of Cardiovascular Science, University College London London UK

## Abstract

**Purpose:**

To investigate whether synthetically generated fractal data can be used to train deep learning (DL) models for dynamic MRI reconstruction, thereby avoiding the privacy, licensing, and availability limitations associated with cardiac MR training datasets.

**Methods:**

A training dataset was generated using quaternion Julia fractals to produce 2D + time images. Multi‐coil MRI acquisition was simulated to generate paired fully sampled and radially undersampled k‐space data. A 3D UNet deep artifact suppression model was trained using these fractal data (F‐DL) and compared with identical models trained on natural videos (NV‐DL) and cardiac MRI data (CMR‐DL). All models were evaluated on prospectively acquired radial real‐time cardiac MRI from 10 patients. Reconstructions were compared against compressed sensing (CS) and low‐rank deep image prior (LR‐DIP). All reconstructions were ranked for image quality, while ventricular volumes and ejection fraction were compared with reference breath‐hold cine MRI.

**Results:**

There was no significant difference in qualitative ranking between F‐DL, NV‐DL, and CMR‐DL (*p* > 0.75), while both outperformed CS and LR‐DIP (*p* < 0.05). Ventricular volumes and function derived from F‐DL were similar to CMR‐DL, showing no significant bias and acceptable limits of agreement compared to reference cine imaging.

**Conclusion:**

A DL model trained using synthetic fractal data reconstructed real‐time cardiac MRI with image quality and clinical measurements comparable to a model trained on true cardiac MRI data. Fractal training data provide an open, scalable alternative to clinical datasets and may enable development of more generalisable DL reconstruction models for dynamic MRI.

## 1 Introduction

Real‐time MRI enables assessment of dynamic changes without physiological gating but requires significant data undersampling to ensure sufficient spatiotemporal resolution. Compressed Sensing (CS) is widely used to reconstruct artifact‐free images from undersampled real‐time data [1], but is limited by time‐consuming iterative reconstructions. More recently, Deep Learning (DL) reconstruction methods have demonstrated faster inference and superior image quality compared to CS [2].

One DL approach for rapid reconstruction of real‐time MRI data is deep artifact suppression (DAS), a “post‐processing” method that has strong performance when combined with non‐Cartesian trajectories. However, DAS is limited by the need for application‐specific MRI training datasets, which can be difficult to obtain and share. It has been previously shown that open‐source videos can be used to train DL‐based MRI reconstructions [3, 4], but their use is restricted by: (i) Clinical skepticism toward DL reconstructions trained on highly out‐of‐distribution content (e.g., animals or landscapes); and (ii) Licensing restrictions on video datasets, which may complicate commercial deployment.

To address these limitations, we propose Julia set fractals as a synthetic source of training data. Although nonanatomical, these fractals exhibit the multi‐scale spatial complexity and smooth temporal dynamics characteristic of cine MRI. In addition, our previously described natural video pipeline can be readily applied to fractal data [3], providing a scalable, open‐source source of training data free from copyright and privacy constraints.

The aims of this proof‐of‐concept study are: (i) To demonstrate the feasibility of generating a synthetic fractal dataset; (ii) To use this data to train a DAS model for dynamic MRI reconstruction; (iii) To evaluate the approach on prospectively acquired radial real‐time cardiac MRI data; and (iv) To compare performance against other reconstruction methods, including DAS trained on natural videos and cardiac MRI data, CS and Deep Image Prior (DIP).

## 2 Methods

### 2.1 Fractal Training Data

Synthetic Julia set data were generated by: (i) Producing Julia set fractals in quaternion space; (ii) Converting them to complex‐valued dynamic images; and (iii) Simulating multi‐coil acquisition to obtain k‐space data. An overview is shown in Figure 1 with all code available at: https://github.com/mrphys/Image_Reconstruction_Fractal.

**FIGURE 1:**
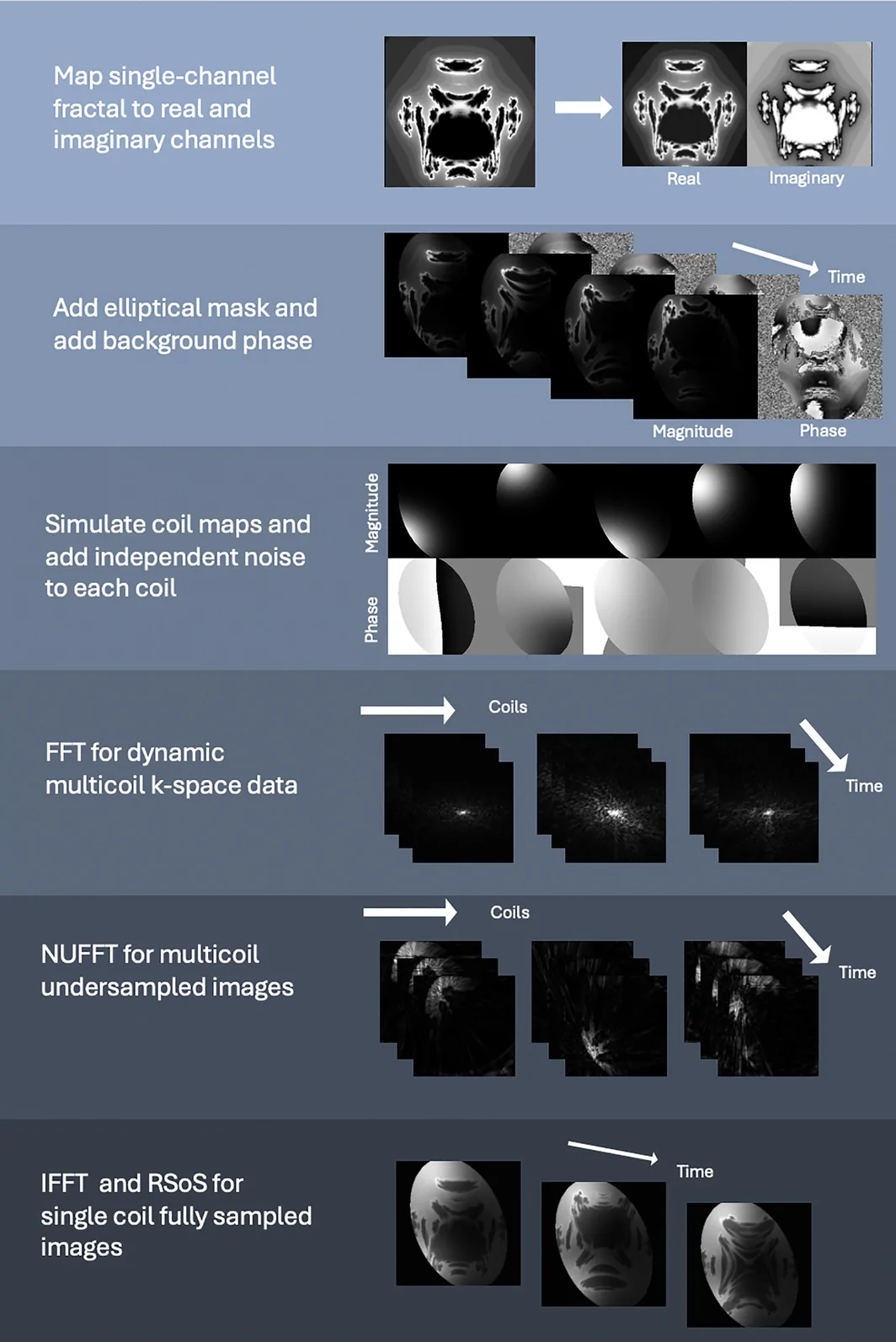
Process for creating multi‐coil complex data from fractals: (i) Map single‐channel fractals to 2 channels (real and imaginary) using two sine waves; (ii) Simulate shape of a body using elliptical masking and add low‐frequency background phase; (iii) Simulate coils using Gaussian intensity distributions and add independent noise to each coil; (iv) Apply a fast Fourier Transformation to acquire data in k‐space; (v) Apply NUFFT with sampling trajectory to acquire undersampled images used as inputs for training; and (vi) Apply inverse fast Fourier Transformation, and Root Sum‐of‐Squares to get single coil fully sampled images used as ground truth for supervision.

#### 2.1.1 Julia Fractal Generation

A Julia fractal is a pattern generated by repeated application of the nonlinear complex‐valued function: $f\left(z\right)=z^{2}+c$, where *z* is a spatial coordinate in a complex domain, and *c* is a user‐defined parameter that controls the structure of the fractal. Although Julia fractals are usually generated in the 2D complex domain, simulation of real‐time MRI data requires two spatial and one temporal dimension. Thus, we generated Julia fractals in quaternion space (4D complex domain) and extracted a 3D sub‐volume to represent 2D + time.

A single Julia fractal was generated via the following steps:

1. A 4D grid with uniform grid spacing that reflects the dimensions of the real data (X,Y,Z,T)∈[−1,1]×[−1,1]×[−0.5,0.5]×[−0.2,0.2] was created, and a c value was selected (see below).
2. For each grid‐point with coordinates (x,y,z,t)∈(X,Y,Z,T):
  - The quaternion $q_{0}$ was initialised to the coordinate vector ($q_{0}=x+yi+zj+tk$), where *i, j, k* are imaginary basis units of the quaternion.
  - The recurrence relationship: $q_{n}=q_{n-1}^{2}+c$, was then iteratively applied, starting with *n* = 1.
  - Iteration was terminated when $\left|q_{n}\right|>4.0$ (empirically determined threshold) or after a maximum of 50 iterations, and the corresponding grid point was assigned the value of n (i.e., the number of iterations to divergence).

#### 2.1.2 Selection of Parameter ‘c’ Using the Mandelbrot Set

A potentially unlimited number of distinct Julia fractals can be generated by varying c. However, most values produce blank or noise‐like images. Thus, we used an empirical strategy to identify values of c which yielded structurally intricate patterns. Specifically, we observed that Julia fractals where the value of the central point had an iteration count between 10 and 30 produced the richest structures. Furthermore, these fractals could be efficiently identified using the Mandelbrot set, which is essentially a catalogue of Julia fractal behavior. The recurrence for the Mandelbrot is the same as the Julia fractal, but $q_{0}$ is fixed for all grid‐points ($q_{0}=0+0i+0j+0k$), while c is set to each grid coordinate (c=x+yi+zj+tk). Grid‐points in the Mandelbrot set with values between 10 and 30 are identified and the associated *c* values are used to generate the Julia fractals (Video S1 compares Julia fractals generated using different *c* parameters).

#### 2.1.3 Conversion to Complex‐Valued Dynamic Images

From each 4D fractal, we extracted the central slice in the z‐axis to represent our 2D + time data. To simulate complex‐valued image data, real and imaginary channels were generated using the equations below:

Re(x)=1281+sin2πf1*norm(x)+φ1

Im(x)=1281+sin2πf2*norm(x)+φ2

where $f_{1}$ was fixed at 0.25 Hz, $f_{2}$ was uniformly sampled from 0.25 to 1 Hz and the phases $\varphi_{1}$ and $\varphi_{2}$ were randomly sampled between 0 and 2π radians and norm is 0–1 normalization. This formulation explicitly couples the magnitude to the phase such that temporal changes are minimal in static portions of the image and larger in areas with more movement. Each channel was Gaussian blurred (*σ* = 0.2–0.4) to suppress fine fractal detail, then sharpened using unsharp masking (Gaussian *σ* = 0.1, *α* = 50) to avoid overly smooth edges.

#### 2.1.4 Simulation of Multi‐Coil MR Data

We follow the method previously applied to natural video data [3] to obtain multi‐coil data consisting of the following steps:

1. A randomly scaled and rotated elliptical mask was applied to simulate body anatomy.
2. Background phase was simulated using a randomly generated 6 × 6 matrix, upsampled to image resolution and kept constant over time.
3. Synthetic coil sensitivity maps were generated using 2D Gaussian functions with random positions, widths, phases, and intensities.
4. Multi‐coil data were created by applying the coil maps to the complex images and adding complex white Gaussian noise to achieve an SNR of 12–22 dB.
5. Fully sampled Cartesian multi‐coil k‐space was generated using the Fast Fourier Transform (FFT).

Using the above steps, a dataset of 692 paired fractal image sets was generated to match the size of the available cardiac MRI training dataset [3].

### 2.2 Cardiac MRI and Natural Video Training Data

We used previously described [3] datasets for the natural video and cardiac MR training.

The cardiac MR dataset included 692 electrocardiogram‐triggered, Cartesian balanced steady state free precession (bSSFP) CINE multi‐coil raw acquisitions obtained in seven cardiac orientations (see Text S1). The raw data were acquired with 2 × undersampling, with 44 reference lines, allowing fully‐sampled multi‐coil Cartesian k‐space data to be reconstructed using GRAPPA.

The natural video dataset was adapted from the Inter4k [5] dataset, with random RGB channels acting as real and imaginary values for complex data. The data was then processed in the same way as the fractals to produce multi‐coil dynamic k‐space.

### 2.3 Training the Deep Artifact Suppression Model

DAS models were separately trained on fractal, natural video and cardiac MRI data. The architecture was based on a previously described [3] 3D UNet [6] that takes multi‐coil, complex, 2D + time data as input and outputs single‐channel, magnitude, 2D + time data (Full architecture details in Figure S1).

All data were processed in a similar fashion to create paired training data that mirrored the prospective data. The fully‐sampled Cartesian multi‐coil k‐space data was compressed to 10 coils using singular value decomposition and resampled onto 13 sorted golden angle radial spokes, rotated every time frame. This radially undersampled multi‐coil k‐space data was then nonuniformly fast Fourier transformed (NUFFT) using the cuFINUFFT algorithm [7]. The resultant multi‐coil, complex 2D + time image data was normalized so that the coil combined magnitude image data was in the range (0, 1). This data was input into the 3D UNet with the ground truth target being the normalized coil combined (root‐sum‐of‐squares), 2D + time, magnitude data derived from the fully‐sampled k‐space data.

Three separate 3D UNet models were trained with a training/validation/test split of 519/69/104 for 300 epochs with the Structural Similarity Index Measure (SSIM) [8] loss and Adam [9] optimizer with learning rate set to $10^{-4}$. We chose to use SSIM loss instead of L1 or L2 because SSIM emphasizes perceptual and structural similarity rather than pixel‐wise differences and was empirically found to yield less noisy reconstructions. Training was performed on a single NVIDIA GeForceRTX 4090 and the training code and pre‐trained networks are available at: https://github.com/mrphys/Image_Reconstruction_Fractal.

### 2.4 Prospective Data Collection

Ten patients (full details in Text S2) referred for cardiac MRI were imaged on a 1.5 T MRI scanner (Avanto, Siemens Healthineers, Germany). Exclusion criteria were inability to breath‐hold and arrhythmia. This study was approved by the UK Health Research Authority and written informed consent was obtained in all subjects (ref. REC19/LO/1561).

A real‐time, sorted golden‐angle radial, bSSFP short‐axis (SAX) stack was acquired during free breathing with each slice collected over two R–R intervals, one for steady‐state preparation and one for data acquisition (details in Text S3). The temporal resolution was ∼35 ms (13 spokes/frame) with the number of frames depending on R‐R interval. These data were reconstructed using DL models trained with fractals (F‐DL), natural videos (NV‐DL), and Cardiac MRI data (CMR‐DL). We also compared these supervised DL methods with (i) low‐rank deep image prior (LR‐DIP) [10], a state‐of‐the‐art unsupervised DL approach requiring no training data, and (ii) conventional compressed sensing (CS) with temporal total variation regularization (5 × 10^−4^).

For LR‐DIP, subspace ranks of 10, 15, and 20 were evaluated on one prospective reconstruction. Rank 10 produced step‐like motion, while rank 20 showed flickering artifacts. Consequently, a rank of 15 was selected, using default parameters and 2000 training epochs per series [10]. Both methods reconstructed multi‐coil 2D + time images directly from undersampled k‐space, with coil combination performed after reconstruction. All reconstructions were temporally interpolated to 30 frames for analysis. In addition, reference‐standard quantification of ventricular volumes was derived from a SAX, breath‐hold bSSFP CINE stack (details in Text S4).

### 2.5 Prospective Data Analysis

Qualitative image evaluation: The mid‐ventricular slice from all five reconstruction methods (F‐DL, NV‐DL, CMR‐DL, LR‐DIP and CS) was assessed by two CMR specialists (MQ, RV). All reconstructions were presented in a single CINE clip arranged in a 5 × 1 side‐by‐side layout, with the order randomly shuffled, blinded to the observers. The observers ranked the images against each other based on subjective image quality, from best (1) to worst (5) with the possibility of tied ranks. Observers were instructed to consider multiple factors in their assessment, including noise level, residual artifacts, sharpness, preservation of anatomical detail, and cardiac motion when determining the final ranking (example in Video S2).

Quantitative image evaluation: Reconstruction quality was assessed using contrast‐to‐noise ratio (CNR), edge sharpness (ES), and the temporal standard deviation of ES. Contrast was calculated as the difference in mean signal intensity between the LV blood pool and myocardium and was normalized by the temporal standard deviation of lung signal intensity to produce CNR. ES was measured across the LV blood pool–myocardium interface using parametric modeling [11], while the temporal standard deviation of ES quantified sharpness consistency over time. All measurements were repeated three times at different boundary locations and averaged.

Comparison of Volumes and Function: Left ventricular and right ventricular (RV) end‐diastolic volume (EDV), end‐systolic volume (ESV) and ejection fraction (EF) were calculated for each reconstruction and compared to reference‐standard breath‐hold CINE data. For all data, preliminary segmentations (all slices and frames) were performed using a pretrained nnUNet model [12] that previously won the M&Ms challenge [13]. These were manually corrected by a CMR expert (VM) using an open‐source in‐house application (Available at: https://github.com/Ti-Yao/Roundel_Registry) to obtain final segmentation masks. Papillary muscles and trabeculae were included in the blood‐pool.

### 2.6 Statistics

For each reconstruction type, average qualitative rankings were compared using Friedman's test with post hoc Nemenyi analysis. CNR and ES were compared using repeated‐measures ANOVA. Clinical metrics were compared with reference‐standard breath‐hold data using Bland–Altman analysis, paired *t*‐tests with Holm correction, and two‐way random‐effects Intraclass Correlation Coefficient (ICC(2,1)). Statistical significance was defined as *p* < 0.05. Differences in ICC were assessed using nonparametric bootstrap resampling (5000 iterations), with significance inferred when the 95% confidence interval excluded zero.

## 3 Results

### 3.1 Fractal Generation and Training of Deep Artifact Suppression

Each multi‐coil, complex, 2D + time fractal dataset took 11.5 s to generate (∼134 min to generate the whole fractal dataset, with 692 examples). The training time for F‐DL, NV‐DL and CMR‐DL 3D UNets was 8 h. Results of evaluation on simulated data are shown in Table S1. F‐DL and NV‐DL reconstructions were inferior to CMR‐DL on the simulated data, but this is to be expected since this data lies within the training distribution of the CMR‐DL model but represents an out‐of‐distribution setting for F‐DL and NV‐DL.

### 3.2 Prospective Study

#### 3.2.1 Inference Time

CMR‐DL, NV‐DL, and F‐DL had identical inference times of ∼400 ms per slice (all frames), resulting in a total reconstruction time of ∼4–6 s for all SAX slices per subject. The CS reconstruction took ∼15 s per slice (all frames), resulting in ∼2–3 min for the whole SAX stack. Finally, LR‐DIP reconstruction required ∼30 min per slice (all frames), leading to a total reconstruction time of ∼5–8 h.

#### 3.2.2 Image Quality

Representative F‐DL, NV‐DL, CMR‐DL, LR‐DIP and CS reconstructions of a mid‐ventricular SAX slice are shown in Figure 2 (Video S3). NV‐DL, F‐DL and CMR‐DL received the highest qualitative scores, with no differences between them (*p* > 0.750), and all outperforming LR‐DIP and CS (*p* < 0.050; Table 1). No unrealistic structures were identified in the F‐DL or NV‐DL reconstructions. However, all supervised DL methods exhibited spatiotemporal smoothing, consistent with the inductive bias of convolutional networks.

**FIGURE 2:**
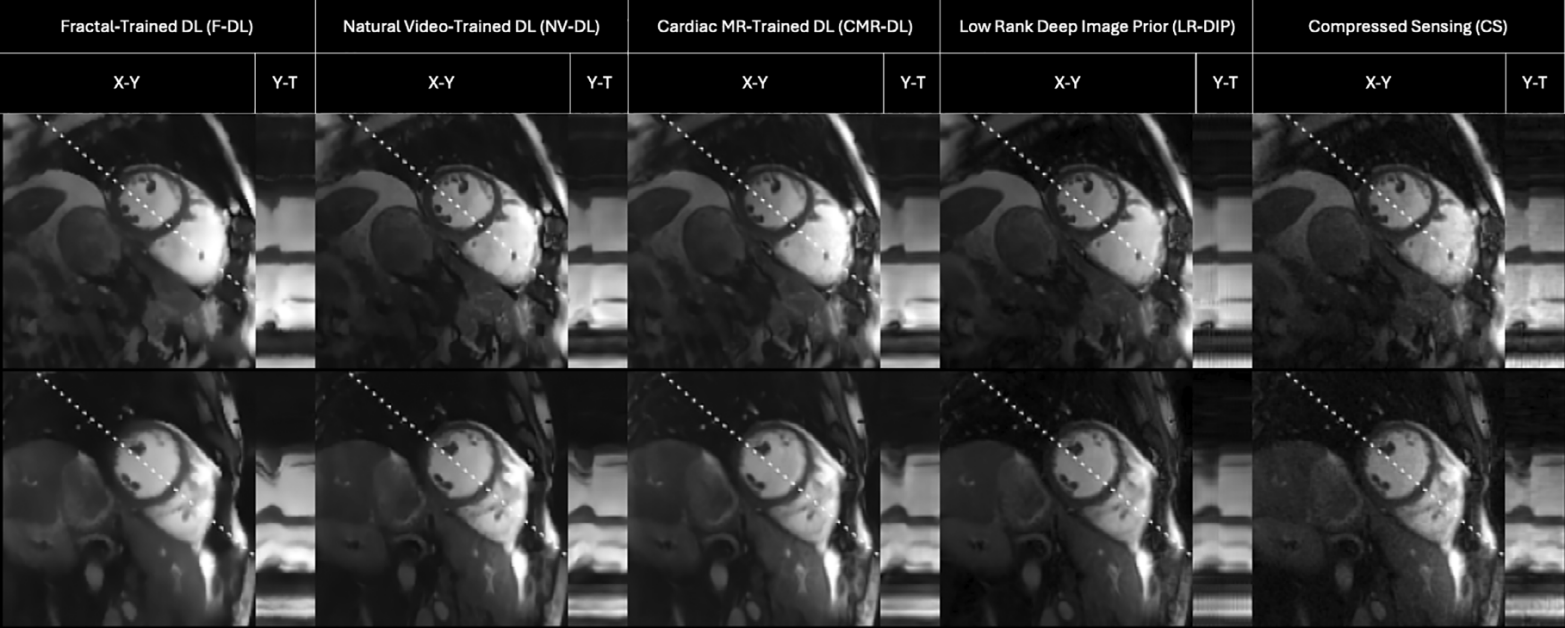
Comparison of reconstructions of real‐time MRI data using the five methods (left to right: F‐DL, NV‐DL, CMR‐DL, LR‐DIP, CS) with X‐Y plots of the mid‐ventricular short‐axis slice at end‐diastole and Y‐T plots of a line across the ventricular septum through time.

**TABLE 1:** Image quality comparison of real‐time data from all five reconstruction methods (F‐DL, NV‐DL, CMR‐DL, LR‐DIP, and CS) and reference‐standard breath‐hold (BH) images, with edge sharpness (ES) in mm^−1^, temporal STD ES in mm^−1^, contrast, contrast‐to‐noise ratio (CNR) and subjective image quality ranking (1 = best, 5 = worst).

Reconstruction	Subjective image quality ranking	Edge sharpness (ES) (mm^−1^)	Temporal STD ES (mm^−1^)	Contrast	CNR
F‐DL	2.4 ± 0.8	0.235 ± 0.068*	0.0525 ± 0.0234	0.219 ± 0.079	154.9 ± 90.2
NV‐DL	1.4 ± 0.8	0.262 ± 0.081	0.0745 ± 0.0336^†^	0.181 ± 0.064^†^	147.5 ± 111.6
CMR‐DL	2.2 ± 0.7	0.247 ± 0.072	0.0627 ± 0.0269	0.171 ± 0.067^†^	102.5 ± 86.4^†*^
LR‐DIP	4.6 ± 0.5^†*★^	0.220 ± 0.072^*★°^	0.0794 ± 0.0970	0.164 ± 0.065^†*^	44.8 ± 33.8^†*★^
CS	4.2 ± 0.9^†*★^	0.240 ± 0.061	0.0721 ± 0.0322^†^	0.165 ± 0.064^†*^	25.9 ± 8.2^†*★▲^

*Note:* The superscripts †, *, ★, ▲, ° indicate statistically significant inferiority to F‐DL, NV‐DL, CMR‐DL, LR‐DIP, and CS, respectively.

There was no significant difference in ES between F‐DL, CMR‐DL and CS reconstructions, although CMR‐DL and CS outperformed LR‐DIP (*p* < 0.002), and NV‐DL outperformed F‐DL and LR‐DIP (*p* < 0.005). F‐DL had the lowest temporal variability in ES and was significantly more consistent than NV‐DL and CS (*p* < 0.004). F‐DL also achieved the highest contrast, exceeding all other methods (*p* < 0.001). For CNR, there was no difference between F‐DL and NV‐DL (*p* = 0.651), whereas all other pairwise comparisons were significant (*p* < 0.010), with performance ranked as CMR‐DL > LR‐DIP > CS. However, CNR estimates should be interpreted cautiously, as noise estimation based on local pixel variability may favor oversmoothed DL reconstructions.

#### 3.2.3 Volumes and Function

The Bland–Altman plots showing agreement in clinical metrics from all reconstruction methods compared to reference‐standard breath‐hold data are shown in Figure 3, with bias and limits of agreement reported in Table 2. For the LV/RV volumes and EF, there were no significant biases (*p* > 0.100) and acceptable limits of agreement for all reconstructions apart from LR‐DIP, which showed significantly higher bias for LV EF (*p* < 0.05) compared to other reconstructions and wider limits of agreement across all measurements.

**FIGURE 3:**
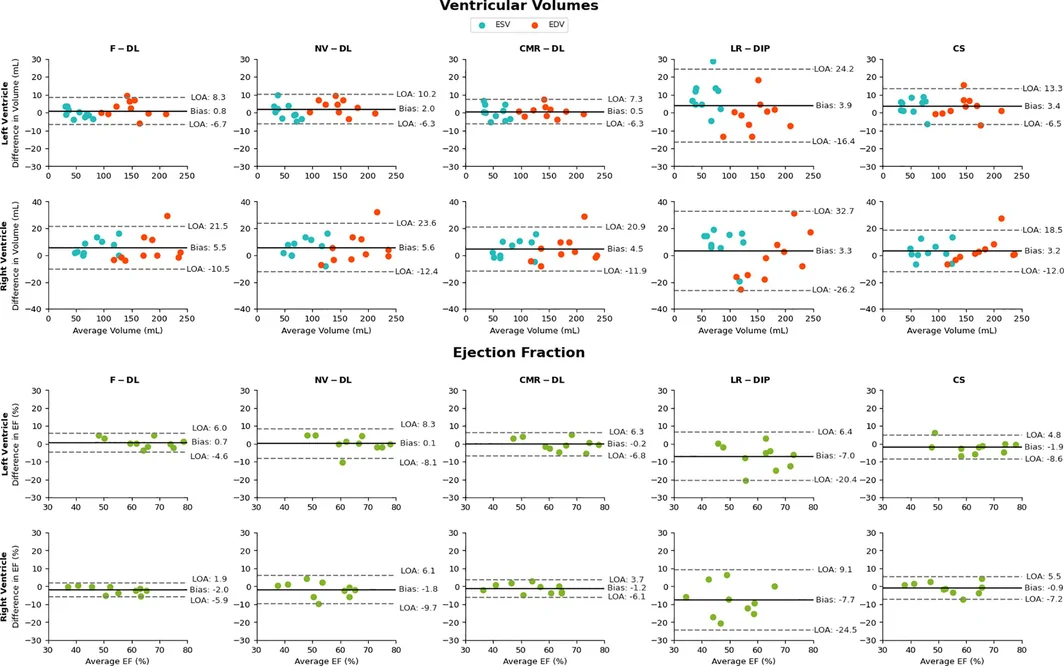
Bland–Altman plots of real‐time data with all five reconstructions (F‐DL, NV‐DL, CMR‐DL, LR‐DIP, CS) compared to reference‐standard breath‐hold data for measurements of end‐systolic volume (ESV), end‐diastolic volume (EDV), and ejection fraction (EF) for the left ventricle (LV) and right ventricle (RV).

**TABLE 2:** Clinical metric comparison of measurements from real‐time data with all five reconstructions (F‐DL, NV‐DL, CMR‐DL, LR‐DIP, and CS) with bias, limits of agreement (LOA) and intraclass correlation coefficient (ICC) between reconstructions and reference‐standard breath‐hold data.

Metric	Reconstruction	Left ventricle			Right ventricle		
		Bias	LOA	ICC	Bias	LOA	ICC
End‐systolic volume	F‐DL	−0.49 mL	4.43 to −5.4 mL	0.99 (0.97 to 1)	6.32 mL	17.38 to −4.75 mL	0.96 (0.62 to 0.99)
	NV‐DL	0.62 mL	8.75 to −7.52 mL	0.96 (0.85 to 0.99)	6.32 mL	25.64 to −13.00 mL	0.92 (0.7 to 0.98)
	CMR‐DL	0.3 mL	7.93 to −7.33 mL	0.98 (0.92 to 0.99)	4.74 mL	17.5 to −8.03 mL	0.97 (0.83 to 0.99)
	LR‐DIP	9.44 mL	26.12 to −7.33 mL	0.82 (0.13 to 0.96)	9.00 mL	29.25 to −11.26 mL	0.89 (0.44 to 0.97)^†★^
	CS	3.78 mL	12.27 to −4.72 mL	0.96 (0.74 to 0.99)	2.92 mL	15.43 to −9.6 mL	0.97 (0.9 to 0.99)
End‐diastolic volume	F‐DL	2.15 mL	10.78 to −6.49 mL	0.99 (0.96 to 1)	4.67 mL	24.23 to −14.88 mL	0.97 (0.87 to 0.99)
	NV‐DL	3.23 mL	13.06 to −4.79 mL	0.98 (0.88 to 1)	4.08 mL	26.71 to −18.55 mL	0.96 (0.85 to 0.99)
	CMR‐DL	0.68 mL	6.51 to −5.15 mL	0.99 (0.98 to 1)	4.28 mL	23.57 to −15.02 mL	0.97 (0.88 to 0.99)
	LR‐DIP	−1.62 mL	15.87 to −19.10 mL	0.97 (0.88 to 0.99)	−2.46 mL	30.23 to −35.16 mL	0.94 (0.77 to 0.98)
	CS	3.05 mL	14.14 to −8.03 mL	0.98 (0.93 to 1)	3.53 mL	21.1 to −14.05 mL	0.97 (0.91 to 0.99)
Ejection fraction	F‐DL	0.69%	6% to −4.62%	0.96 (0.86 to 0.99)	−2.02%	1.9% to −5.95%	0.96 (0.67 to 0.99)
	NV‐DL	−0.10%	8.01% to −9.14%	0.91 (0.68 to 0.98)	−2.46%	7.74% to −12.67%	0.84 (0.51 to 0.96)
	CMR‐DL	−0.23%	6.35% to −6.8%	0.95 (0.8 to 0.99)	−1.18%	3.72% to −6.08%	0.96 (0.86 to 0.99)
	LR‐DIP	−7.00% ^†°*★^	6.38% to −20.39%	0.61 (0.04 to 0.89)^†°*★^	−7.72%	9.09% to −24.52%	0.51 (0.06 to 0.85)^†°*★^
	CS	−1.91%	4.77% to −8.6%	0.93 (0.74 to 0.98)	−0.85%	5.48% to −7.18%	0.95 (0.81 to 0.99)

*Note:* The superscripts †, *, ★, ° indicate statistically significant inferiority to F‐DL, NV‐DL, CMR‐DL, and CS, respectively. Measurements included end‐systolic volume (ESV), end‐diastolic volume (EDV) and ejection fraction (EF) of the left ventricle (LV) and right ventricle (RV).

ICCs for RV/LV volumes and function were high for all reconstructions apart from LR‐DIP when compared to reference‐standard breath‐hold data (Table 2). LR‐DIP reconstructions had significantly lower ICCs for LV EF, as well as RV ESV and RV EF.

## 4 Discussion

The main finding of this proof‐of‐concept study is that a DL‐based reconstruction model can be successfully trained using synthetic fractal data. Importantly, this model achieved image quality and clinical measurements comparable to a model trained on real CMR data.

Although open‐source CMR repositories are becoming increasingly available (e.g., CMRXrecon [14]), our fractal‐based framework offers several advantages. First, it enables generation of virtually unlimited synthetic k‐space data, which may benefit data‐intensive architectures, although this requires further investigation. Second, fractals are not anatomy‐specific and can be applied to diverse real‐time applications. Third, fractals naturally support training for 3D + t imaging applications (e.g., 3D cine), where training data are limited.

In our prospective real‐time MRI experiments, F‐DL, NV‐DL, and CMR‐DL reconstructions demonstrated comparable image quality, consistent with our previous findings [3]. This likely reflects DAS primarily learning local image features, which are shared between real and synthetic data. Furthermore, quantitative analysis showed that ventricular volumes and functional parameters derived from F‐DL reconstructions were in good agreement with reference‐standard breath‐hold CINE imaging.

We also compared supervised DL approaches with alternative reconstruction techniques, including LR‐DIP and conventional CS. Overall, supervised DL methods slightly outperformed both LR‐DIP and CS. More notably, reconstruction times for LR‐DIP and CS were substantially longer, with LR‐DIP requiring hours to reconstruct a SAX stack. Although newer iterative self‐supervised methods offer improved reconstruction speeds [15], they may also benefit from pretraining using synthetic fractal data.

This proof‐of‐concept study has several limitations. Only a single sampling pattern and DL architecture were evaluated, and future work should assess generalisability across sampling trajectories and reconstruction methods. This is pertinent, as iterative DL approaches incorporating data‐consistency may reduce the subtle spatiotemporal over‐smoothing observed with simple DL artifact suppression. Additionally, our fractal generation method concentrates intensity variation near fractal boundaries, which may not fully represent anatomical image features. Alternative generation models (e.g., Perlin noise) could provide more spatially distributed variation. The framework could also be extended by incorporating recent advances in adapting video data [4] to MRI and synthesizing phase and coil sensitivity maps from magnitude‐only images [16, 17].

Finally, systematic ablation studies are needed to evaluate the effects of fractal parameterisation, dynamic range, and training set size, enabling optimisation of the framework. In addition, larger and more diverse patient cohorts are required for comprehensive clinical validation.

## 5 Conclusion

We developed and demonstrated a self‐generated fractal data framework for training a DL model for dynamic MRI reconstruction, with strong potential to enable generalisable reconstruction models in the future.

## Funding

This work was supported by the UK Research and Innovation (MR/S032290/1), Engineering and Physical Sciences Research Council (EP/W524335/1).

## Conflicts of Interest

The authors declare no conflicts of interest.

## Supporting information


**Figure S1:** DL Model architecture for F‐DL and CMR‐DL. The model is a 3D UNet with leaky ReLU activations and 3 levels with 64, 96, and 128 filters, respectively.
**Table S1:** Comparison of CMR‐DL, NV‐DL and F‐DL on simulated CMR data reserved for testing from the data acquired for CMR‐DL training. Evaluation Metrics include mean‐squared error (MSE), Structural Similarity Index Measure (SSIM), Peak Signal‐to‐Noise Ratio (PSNR). CMR‐DL's performance was statistically significantly superior to both other models (*p* < 0.001) while NV‐DL and F‐DL did not differ statistically (*p* > 0.05).
**Text S1:** Cardiac MRI training data.
**Text S2:** Demographics of prospective population.
**Text S3:** Undersampled real‐time acquisition details.
**Text S4:** Reference standard acquisition details.

**Video S1:** Comparison of magnitude images generated by varying the ‘c’ value during the Julia fractal‐generation process.

**Video S2:** Example of reconstructions laid out for ranking by observers. Reconstructed videos are unlabelled and displayed in a random order when presented to the observers. The example shows an observer ranking for a set of reconstructions.

**Video S3:** Comparison of reconstructed prospective short‐axis cine images at the mid‐ventricular level from two different subjects, with one subject shown per row. Left to right—fractal‐trained DL (F‐DL), natural video‐trained DL (NV‐DL), cardiac MR‐trained DL (CMR‐DL), low‐rank deep image prior (LR‐DIP), and compressed sensing (CS). Animated X‐Y plots of the mid‐ventricular short‐axis slice and Y‐T plots of a line across the ventricular septum through time.

## Data Availability

The data that support the findings of this study are openly available in Image_Reconstruction_Fractal at https://github.com/mrphys/Image_Reconstruction_Fractal.
