# Supplementary material for: Training Deep Learning Based Dynamic MR Image Reconstruction Using Synthetic Fractals
Source: Magn Reson Med. 2026 Jul 19;96(6):2795–802. doi: 10.1002/mrm.70521 (PMC13619857; doi:10.1002/mrm.70521)
Supplement: Supplementary file 1 [file MRM-96-2795-s001.docx]

**Supplementary Text**

**Text S1:** Cardiac MRI Training Data

The training dataset for the CMR-DL networks comprised 692 electrocardiogram-triggered, breath-held Cartesian balanced SSFP CINE multicoil raw data acquisitions. The dataset included seven imaging orientations: short axis, four-chamber, three-chamber, two-chamber, right ventricular long axis, right ventricular outflow tract, and pulmonary artery views. These data were obtained from a diverse adult patient population referred for routine cardiovascular MRI examinations, including evaluation of ischemia, cardiomyopathy, and pulmonary hypertension (age: 58.7 ± 16.0 years; weight: 77.4 ± 18.6 kg; male/female: 56/36).

The raw data were acquired with two-fold undersampling, using a nominal matrix size of 224 × 272 and 44 autocalibration lines, and were reconstructed using GRAPPA to generate fully sampled reference Cartesian multicoil k-space data. From this reference data, both undersampled input images and high-quality target output images were derived.

The target magnitude images were generated by applying fast Fourier transformation (FFT) to the fully sampled Cartesian multicoil k-space data, followed by coil combination using the root sum-of-squares (RSS) method. The resulting ground-truth images were normalised to a range between 0 and 1.

**Text S2:** Demographics of prospective population

The prospective data were obtained from a diverse adult patient population referred for routine cardiovascular MRI examinations, including evaluation of cardiac amyloidosis, cardiac Fabry, myocarditis and arrhythmogenic right ventricular cardiomyopathy (age: 61.2 ± 20 years; weight: 71.9 ± 11.9 kg; male/female: 7/3).

**Text S3:** Undersampled Real-time Acquisition Details

The undersampled real-time data was acquired using a radial sorted golden angle trajectory with the following scan parameters: slice thickness= ~7mm, slice spacing = ~10mm, spatial resolution = ~1.75x1.75mm, matrix size = 256x256, FOV = ~450x450mm, TE/TR = ~1.4/2.8ms, temporal resolution = ~35ms, flip angle = 70°, bandwidth = 1220Hz/pixel.

**Text S4:** Reference Standard Acquisition Details

The reference-standard breath hold data was acquired using standard BH-bSSFP cine imaging with the following scan parameters: slice thickness = ~7mm, slice spacing = ~10mm, spatial resolution = ~1.5x1.5mm, matrix size = ~256x190, FOV = ~400x300mm, TE/TR = ~1.15/2.3ms, temporal resolution = ~35ms, flip angle = 50°, bandwidth = ~1050Hz/pixel.

**Supplementary Videos**

**Video S1:** Comparison of magnitude images generated by varying the ‘c’ value during the Julia fractal-generation process.

**Video S2:** Example of reconstructions laid out for ranking by observers. Reconstructed videos are unlabelled and displayed in a random order when presented to the observers. The example shows an observer ranking for a set of reconstructions.

**Video S3:** Comparison of reconstructions of prospective short-axis cine images. Left to right - fractal-trained DL (F-DL), natural video-trained DL (NV-DL), cardiac MR-trained DL (CMR-DL), low-rank deep image prior (LR-DIP), and compressed sensing (CS). Animated X-Y plots of the mid-ventricular short-axis slice and Y-T plots of a line across the ventricular septum through time.

**Supplementary Figures**

**Figure S1:** DL Model architecture for F-DL and CMR-DL. The model is a 3D UNet with leaky ReLU activations and 3 levels with 64,96 and 128 filters respectively.

**Supplementary Tables**

**Table S1:** Comparison of CMR-DL, NV-DL and F-DL on simulated CMR data reserved for testing from the data acquired for CMR-DL training. Evaluation Metrics include mean-squared error (MSE), Structural Similarity Index Measure (SSIM), Peak Signal-to-Noise Ratio (PSNR). CMR-DL’s performance was statistically significantly superior to both other models (p<0.001) while NV-DL and F-DL did not differ statistically (p>0.05).

| **Reconstruction** | **SSIM** | **PSNR** | **MSE** |
| --- | --- | --- | --- |
| CMR-DL | 0.88 ± 0.050 | 30.95 ± 3.23 | 0.0011 ± 0.0008 |
| NV-DL | 0.81 ± 0.067 | 26.97 ± 3.13 | 0.0026 ± 0.0024 |
| F-DL | 0.78 ± 0.061 | 24.87 ± 2.68 | 0.0040 ± 0.0028 |
